# Supplementary material for: BIreactive: Expanding the Scope of Reactivity Predictions to Propynamides
Source: Pharmaceuticals (Basel). 2023 Jan 12;16(1):116. doi: 10.3390/ph16010116 (PMC9866037; doi:10.3390/ph16010116)
Supplement: Supplementary file 1 [file pharmaceuticals-16-00116-s001.zip › pharmaceuticals-2070207 - Supplementary material file/Supplementary material file.pdf]

Supporting Information for Reactivity Prediction with BIreactive – Adding Propynamides to the Mix

Markus Rainer Hermann, Christofer Tautermann, Peter Sieger, Marc A. Grundl, Alexander Weber*

Medical Chemistry, Boehringer Ingelheim Pharma GmbH & Co. KG, Birkendorferstr. 65, 88397 Biberach an der Riß, Germany

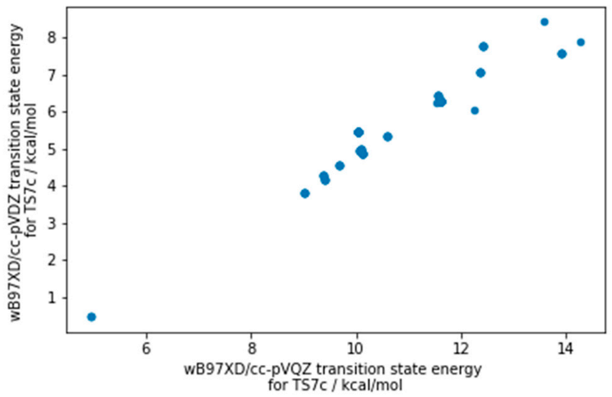

Figure S1: Comparison between the cc-pVDZ and the cc-pVQZ transition state energies for the molecules in the benchmark set. R^2:0.96, MAE: 0.27 kcal/mol.

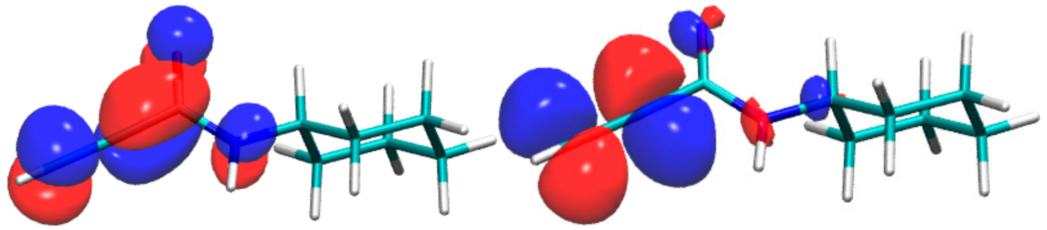

Figure S2: LUMO and LUMO+1 orbitals for 1g (1.01 eV, 2,83 eV).

TS FOR PROPYNAMIDES

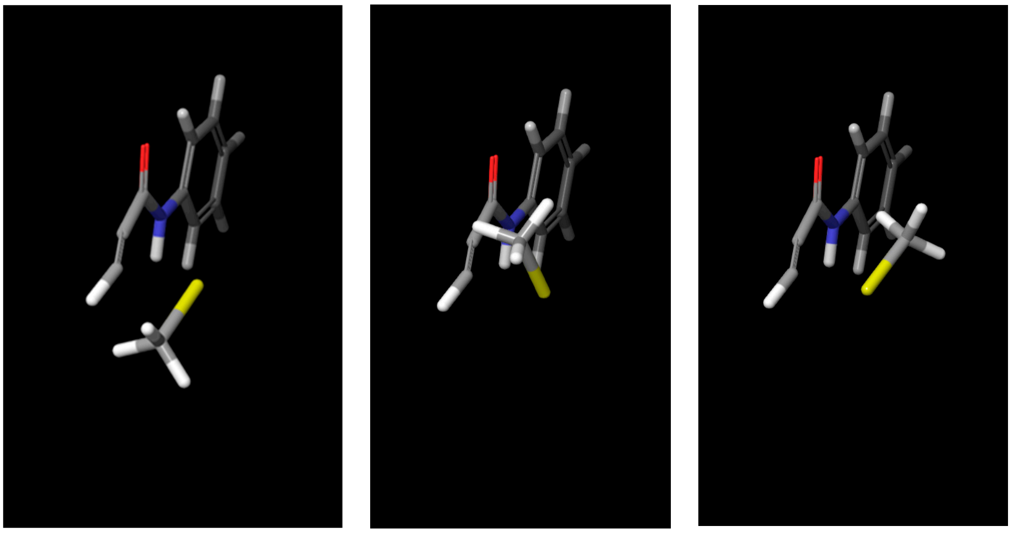

(a) (b) (c)

Figure S3: Geometries of possible transition states compound 1a are shown (1a /1b /1c).

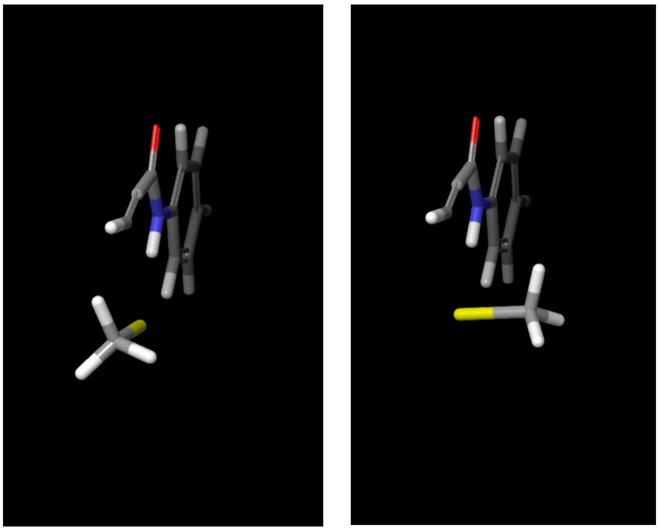

(a) (b)

Figure S4: Geometries of possible transition states compound 1a are shown (2a /2b).

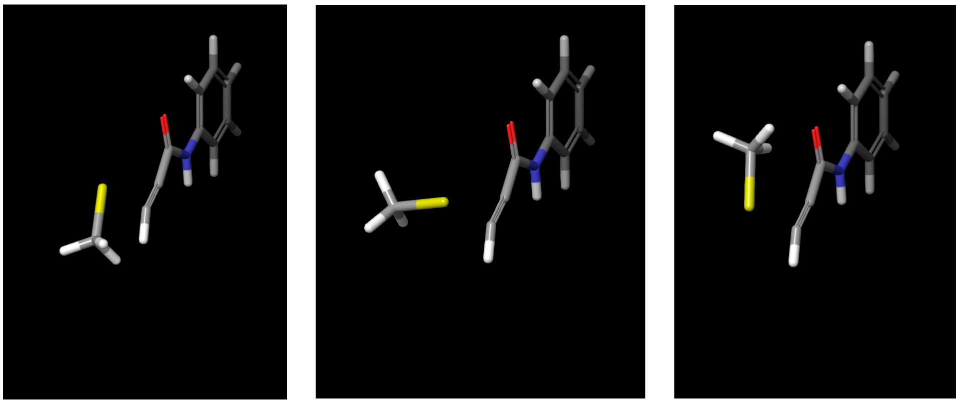

(a) (b) (c)

Figure S5. Geometries of possible transition states compound 1a are shown (3a / 3b / 3c). .

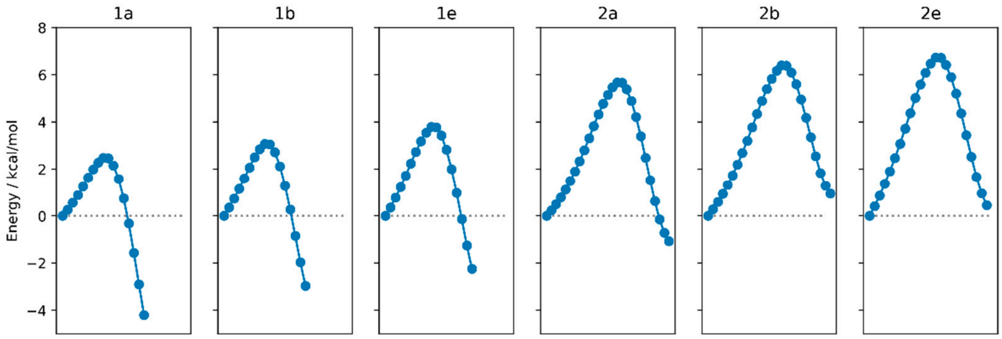

Figure S6: Intrinsic reaction coordinates with electronic energy for the reaction of methanethiolate (CH3S−) and different propynamides. On the x-axis is the reaction coordinate. The highest point in energy is the transition state. Left of the transition state is the way to the educt, while on the right is the progress of the reaction to the product. For the hydrogen substituted propynamides (1a, b, e), the transition state and the educt are lower in energy than for the methyl substituted propynamides (2a, 2b, 2e). This fits with GSH data, that hydrogen substituted propynamides are more reactive than their methyl substituted counterpart.

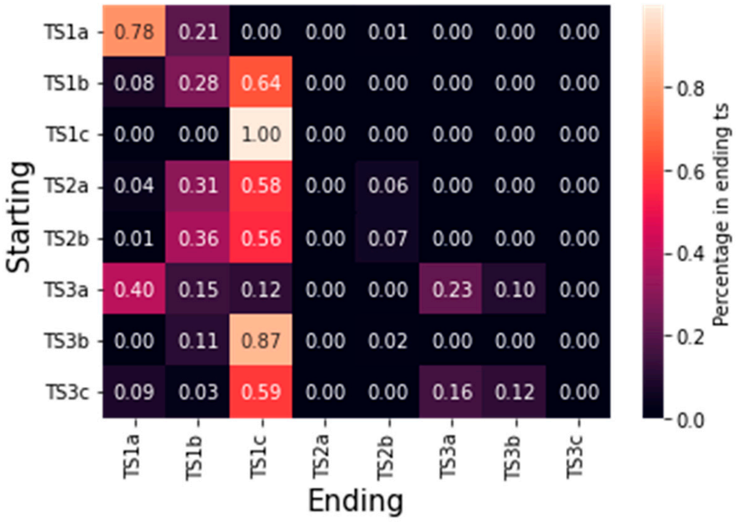

Figure S7: Transition matrix for transition states of propynamides. Shows, how many of the transition states, fall into another transition state (ending) compared from where they started (starting). Transition states are depicted in figures S1-3. The most stable transition state is TS1c. It is most often reached and when started from the arrangement for TS7c, no calculations ended in a different transition state. Only a marginal number of transition states were found where the methanethiolate attacks from the top or bottom.

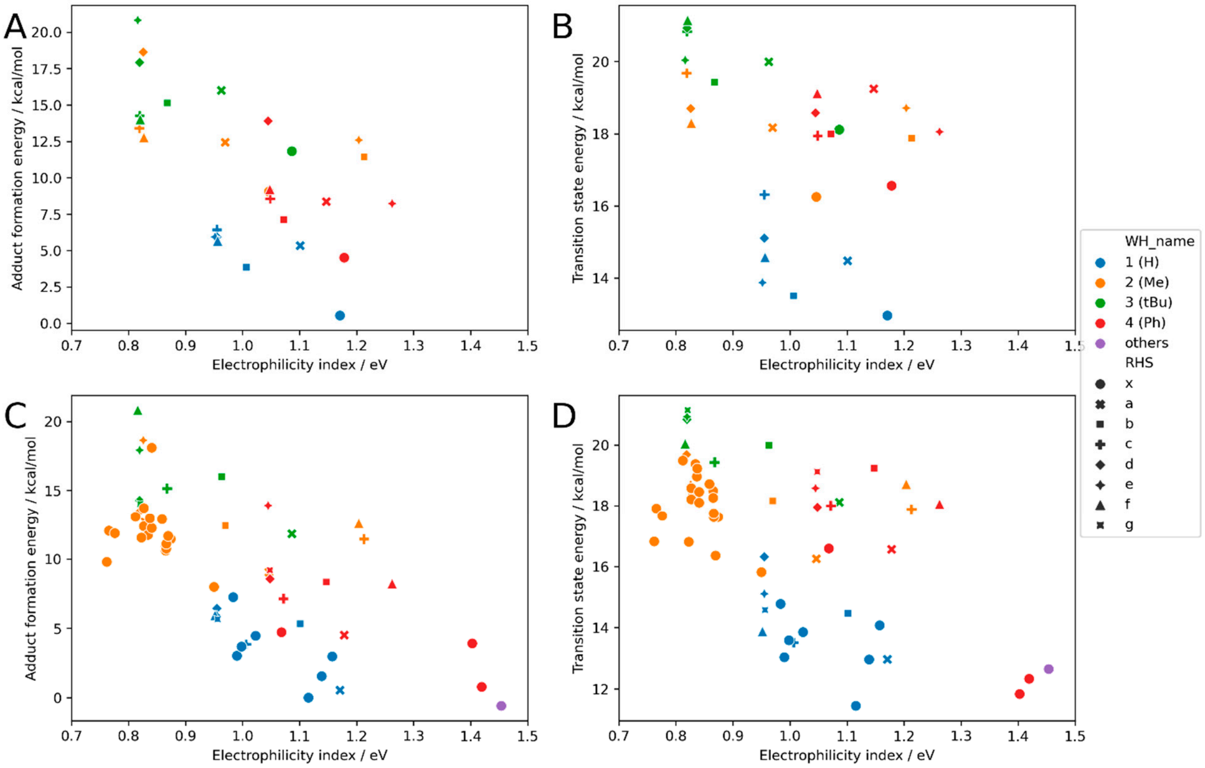

Figure S8: Correlation with the electrophilicity index for the benchmark set (A, B) and the full data set (C, D). R^2*:0.34, 0.15, 0.47 and 0.35.*
